# Efficacy of Telemedicine in Hypertension Care Through Home Blood Pressure Monitoring and Videoconferencing: Randomized Controlled Trial

**DOI:** 10.2196/27347

**Published:** 2021-08-31

**Authors:** Junichi Yatabe, Midori Sasaki Yatabe, Rika Okada, Atsuhiro Ichihara

**Affiliations:** 1 General Incorporated Association TelemedEASE Shinjuku-ku, Tokyo Japan; 2 TM Clinic Nishishinjuku Shinjuku-ku, Tokyo Japan; 3 Department of Endocrinology and Hypertension Tokyo Women’s Medical University Shinjuku-ku, Tokyo Japan

**Keywords:** blood pressure management, digital health, web-based medicine, prospective study, telemonitoring, blood pressure, monitoring, telemedicine, telehealth, efficacy, hypertension, video conference, safety, Japan

## Abstract

**Background:**

The burden of time is often the primary reason why patients discontinue their treatment. Telemedicine may help patients adhere to treatment by offering convenience.

**Objective:**

This study examined the efficacy and safety of telemedicine for the management of hypertension in Japan.

**Methods:**

Patients with uncomplicated hypertension were recruited through web advertising between November 2015 and February 2017. They were then screened, stratified by office systolic blood pressure (SBP), and randomized into two groups: usual care (UC) and telemedicine. The telemedicine group used a 3G network–attached home blood pressure (BP) monitoring device, consulted hypertension specialists from an academic hospital through web-based video visits, and received prescription medication by mail for 1 year. The UC group used the same BP monitoring device but was managed using self-recorded BP readings, which included their diary entries and office BP taken in a community practice setting.

**Results:**

Initial screening was completed by 99 patients, 54% of whom had untreated hypertension. Baseline BP was similar between the groups, but the weekly average SBP at the end of the 1-year study period was significantly lower in the telemedicine group (125, SD 9 mmHg vs 131, SD 12 mmHg, respectively; *P*=.02). SBP in the telemedicine group was 3.4 mmHg lower in the morning and 5.8 mmHg lower in the evening. The rate of SBP control (135 mmHg) was better in the telemedicine group (85.3% vs 70.0%; *P*=.01), and significant adverse events were not observed.

**Conclusions:**

We present evidence suggesting that antihypertensive therapy via home BP telemonitoring and web-based video visits achieve better BP control than conventional care and is a safe treatment alternative that warrants further investigation.

**Trial Registration:**

UMIN-CTR UMIN000025372; https://tinyurl.com/47ejkn4b

## Introduction

Antihypertensive therapy has advanced over the years to enable lowering of blood pressure (BP) in most patients with hypertension if they receive proper treatment. Nevertheless, in Japan, only 12 million of 43 million individuals with hypertension receive treatment and have their BP controlled [1]. This phenomenon, termed the “hypertension paradox,” must be resolved to improve public health [2]. Among the reasons why individuals do not take action to control their hypertension, the burden of time takes precedence for discontinuation or noninitiation of antihypertensive treatment. Telemedicine using internet-based communication may lower the hurdle for starting and adhering to hypertension treatment, eventually leading to the prevention of cardiovascular disease. This approach may also result in higher satisfaction among patients by allowing better use of their time rather than having them spend much of it waiting at clinics or hospitals. Technical difficulty and anxiety associated with telemedicine hence need to be managed sufficiently.

Achieving target BP levels in the treatment of hypertension requires patients’ adherence to and persistence in taking their medication. Self-measurement of home BP (SMBP) helps improve adherence to treatment and aids in BP control [3,4]. Adjusting antihypertensive medication based on self-measured home BP (HBP) for long periods is feasible, and HBP self-monitoring with self-titration of antihypertensive medication in accordance with an individualized predetermined protocol has been reported to result in better BP control than that with usual care (UC) [5].

In Japan, telemedicine without face-to-face communication has been permitted since early 2015. We developed an integrated web-based telemedicine platform to manage appointments, medical care, and payment without patients visiting a clinic. However, clinical evidence concerning the efficacy and safety of telemedicine remains scarce. In this study, we attempted to demonstrate the advantages of telemedicine over traditional care in the management of hypertension. The Paradigm of Antihypertensive Therapy along with Telemedicine Randomized (POATRAND) trial was designed and performed as a prospective, randomized, open-label, 2-arm study of patients with uncontrolled, uncomplicated hypertension to test the effectiveness and safety of BP telemonitoring as well as hypertension telemedicine.

## Methods

### Study Design and Participant Recruitment

The POATRAND trial was a multicenter, open-label randomized controlled trial performed at Tokyo Women’s Medical University Clinic and private clinics in Japan. Potentially eligible participants with hypertension were recruited through web advertising. The inclusion criteria were age over 20 years, elevated systolic BP (SBP) or diastolic BP (DBP), ability to visit the Tokyo Women’s Medical University Clinic for the initial screening, willingness to receive hypertension care through telemedicine, and ability to self-measure HBP. The exclusion criteria were inability to use a smartphone, pregnancy, presence of major cardiovascular events and diabetes mellitus, an estimated glomerular filtration rate lower than 30 mL/min/1.73 m^2^ determined using the modified Modification of Diet in Renal Disease formula for Japanese patients [6], and presence of secondary hypertension excluding primary aldosteronism without surgical indication. The study protocol was approved by the Tokyo Women’s Medical University Research Ethics Committee (approval 160603) and registered in the UMIN Clinical Trials Registry under accession number UMIN000025372. All patients provided their written informed consent to participate in the study.

### Randomization

Potentially eligible patients were invited to Tokyo Women’s Medical University Clinic between November 2015 and February 2017 for screening. After eligibility screening and provision of informed consent, each patient’s office BP was measured with the patient sitting quietly alone in a room. Before measurement, an experienced staff member instructed the patient on the procedure, placed a cuff with an appropriately sized bladder on the patient’s upper left arm, and left the room. BP was measured 3 times at 3-min intervals. Office BP was measured using the same validated BP monitor as the one used for home BP measurements (HEM-7252G-HP; Omron) [7], and the values were not concealed from the participants. At the second baseline visit, screening results were communicated to the participants, and the participants were stratified by the average of the second and third office SBP readings at the first visit and randomly assigned at a 1:1 ratio into the UC or telemedicine group, using an Excel-based random sampling number system.

### Procedures

HBP was measured using a 3G network–equipped automatic sphygmomanometer (HEM-7252G-HP; Omron) [7]. The device was based on the cuff–oscillometric principle and validated to meet the criteria of the Association for the Advancement of Medical Instrumentation. The device recorded and transmitted SBP and DBP values, heart rate, and the date and time of each measurement. Registered patients were instructed on how to use the device and asked to take their HBP reading in a sitting position twice every morning within 1 h of waking before taking a meal or medication and after more than 2 minutes of rest. Participants were also asked to measure their HBP twice every evening before going to bed. The telemedicine group used this device, consulted a physician through web-based visits in consideration of their transmitted BP values, and received prescription medication by mail for 1 year. The UC group used the same BP monitoring device but was managed with actual office visits using self-recorded BP readings, such as their diary entries. After randomization, baseline HBP was measured in both groups without changes in treatment. The standard visit-to-visit interval was set at 6 weeks for the telemedicine group and was unspecified for the UC group, leaving it to the physician’s discretion.

### Primary and Secondary Outcomes

The primary outcome was the average home SBP during the last week of the 12-month study period. Secondary outcomes included other measures of office BP and HBP including morning and evening SBP, DBP, and BP control rates, adverse events (eg, side effects and cardiovascular events), medication prescription (ie, number and defined daily dose), body weight, and laboratory measures.

### Statistical Analysis

We initially estimated that 260 patients were required for screening per group to detect a 4-mmHg difference in home SBP values between the 2 groups, with a 2-sided *P* value of .05 and 80% power. However, the study was prematurely terminated at the end of March 2018 because the health insurance policy in Japan changed, requiring a face-to-face visit at least every 3 months. SPSS (version 21, IBM Corp) was used for statistical analyses. All *P* values were 2-sided, and *P*≤.05 was considered statistically significant. Data are presented as mean (SD) values unless otherwise indicated.

## Results

### Baseline Participant Characteristics

**Figure 1:**
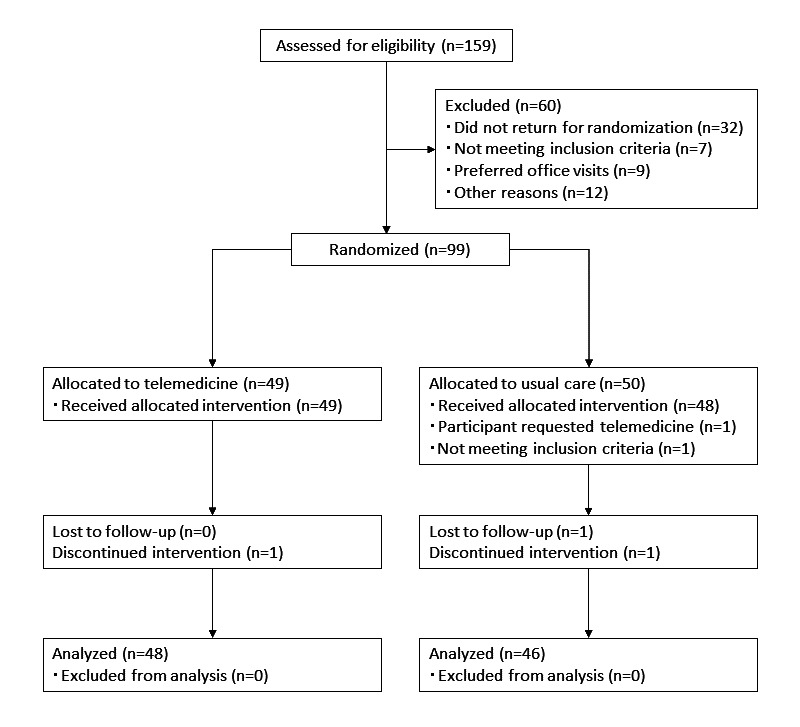
CONSORT (Consolidated Standards of Reporting Trials) flowchart for the study. TM: telemedicine, UC: usual care.

In total, 159 patients were screened for the study (Figure 1). Their baseline characteristics are shown in Table 1. The groups did not show significant differences in age, female-to-male ratio, BMI, home SBP, home DBP, pulse rate, plasma aldosterone concentration, plasma renin activity, estimated glomerular filtration rate, hemoglobin A_1c_ level, and low-density-lipoprotein cholesterol (LDL-C) level. At the end of the study, we assessed 46 individuals from the UC group and 48 from the telemedicine group. Dropout rates and adverse events are discussed subsequently.

**Table 1:** Baseline characteristics of the study participants (N=97).

Parameters	All participants	Usual care group (n=48)	Telemedicine group (n=49)
Female, n (%)	58 (60%)	30 (63%)	28 (57%)
Age (years), mean (SD)	53 (9)	53 (9)	53 (9)
BMI (kg/m^2^), mean (SD)	24.2 (3.9)	24.3 (4.3)	24.2 (3.4)
Home systolic blood pressure (mmHg), mean (SD)	136 (13)	136 (11)	136 (15)
Home diastolic blood pressure (mmHg), mean (SD)	91 (9)	91 (8)	90 (10)
Home pulse rate (bpm), mean (SD)	73 (9)	74 (9)	73 (9)
Antihypertensive treatment, n (%)	55 (57)	29 (60)	26 (53)
Estimated glomerular filtration rate (mL/min/1.73 m^2^), mean (SD)	75.9 (14.0)	77.3 (13.3)	74.4 (14.4)
Low-density-lipoprotein cholesterol (mg/dL), mean (SD)	121.1 (28.8)	119.2 (28.9)	123.0 (28.3)
Hemoglobin A_1c_ (%), mean (SD)	5.7 (0.3)	5.6 (0.3)	5.7 (0.3)
Plasma aldosterone (pg/mL), median (IQR)	155 (119-216)	150 (112-199)	167 (136-232)
Plasma renin activity (ng/mL/h), median (IQR)	1.1 (0.6-1.9)	0.9 (0.5-1.8)	1.2 (0.9-2.0)

### Changes in HBP and Laboratory Data Before and After the Study

Home SBP and DBP significantly decreased after 1 year in both groups (Tables 2 and 3). Plasma renin activity was significantly increased in the UC group (*P*=.02) but not in the telemedicine group.

**Table 2:** Outcomes.

	Usual care group				Telemedicine group				*P* value
	Preintervention (n=48)	Postintervention (n=46)	*P* value	Preintervention (n=49)		Postintervention (n=48)	*P* value		
BMI (kg/m^2^), mean (SD)	24.2 (4.3)	3.9 (4.1)	.86	24.2 (3.4)		23.9 (3.3)	.29	.10	
Home systolic blood pressure (mmHg), median (95% CI)	136 (133-139)	131 (128-134)	.01	136 (132-140)		125 (122-128)	<.001	.02	
Home diastolic blood pressure (mmHg), median (95% CI)	91 (89-93)	87 (85-89)	.02	90 (87-93)		83 (81-85)	<.001	.07	
Home pulse rate (bpm), mean (SD)	74 (9)	71 (7)	.46	73 (9)		71 (8)	.08	.08	
Systolic blood pressure control rate (%)	41.3	70.0	<.001	47.7		85.4	<.001	.01	
Estimated glomerular filtration rate (mL/min/1.73 m^2^), mean (SD)	77.3 (13.3)	76.8 (11.9)	.16	74.4 (14.4)		72.4 (14.4)	.30	.05	
Potassium (mEq/L), mean (SD)	4.1 (0.3)	4.1 (0.3)	.81	4.1 (0.3)		4.1 (0.3)	.65	.93	
Low-density-lipoprotein cholesterol (mg/dL), mean (SD)	119.2 (28.9)	110.3 (22.7)	.53	123.0 (28.3)		123.8 (28.6)	.11	.04	
Hemoglobin A_1c_ (%), mean (SD)	5.6 (0.3)	5.7 (0.3)	.74	5.7 (0.3)		5.7 (0.4)	.60	.53	
Plasma aldosterone (pg/mL), median (IQR)	150 (112-199)	151 (115-226)	.07	167 (136-232)		171 (139-229)	.54	.49	
Plasma renin activity (ng/mL/h), median (IQR)	0.9 (0.5-1.8)	1.1 (0.6-3.6)	.02	1.2 (0.9-2.0)		1.9 (1.1-4.8)	.07	.57	

**Table 3:** Home blood pressure change from baseline till the end of the 1-year study period.

	Usual care group	Telemedicine group	*P* value
Change in systolic blood pressure (mmHg), mean (SD)	–5.4 (11.3)	–9.2 (14.3)	.23
Change in diastolic blood pressure (mmHg), mean (SD)	–3.5 (8.1)	–5.5 (8.7)	.33

### Differences in HBP Between the Participant Groups

The average home SBP during the last week of the study was significantly lower (by 6 mmHg) in the telemedicine group than in the UC group (Table 2). Home DBP after the 1-year study period tended to be lower in the telemedicine group, but the difference was not significant. The average SBP and DBP reached the therapeutic targets of less than 135 mmHg and 85 mmHg, respectively, at the time of measurement in the telemedicine group only.

When morning (4-10:59 AM) and evening (6 PM to 3:59 AM) BPs were analyzed separately (Table 4), the telemedicine group showed significantly lower evening SBP and DBP readings. The average morning SBP and DBP readings were also lower in the telemedicine group, but the difference was not significant.

The number of BP measurements per week for the whole study period was significantly higher in the telemedicine group (17.8, SD 11.5) than in the UC group (12.1, SD 11.0) (*P*=.02).

**Table 4:** Average morning and evening home blood pressure readings during the last week of the 1-year study period.

		Usual care group (n=46), mean (SD)	Telemedicine group (n=48), mean (SD)	*P* value
**Morning**				
	Home systolic blood pressure (mmHg)	134.0 (8.6)	130.6 (10.3)	.09
	Home diastolic blood pressure (mmHg)	90.6 (7.2)	88.3 (7.7)	.14
	Home pulse rate (bpm)	71.6 (8.0)	69.7 (7.5)	.25
**Evening**				
	Home systolic blood pressure (mmHg)	131.6 (8.7)	125.8 (11.5)	.007
	Home diastolic blood pressure (mmHg)	87.2 (7.5)	82.5 (7.5)	.003
	Home pulse rate (bpm)	76.3 (9.4)	74.2 (8.5)	.27

### Clinical Parameters at the End of the Study

At the end of the study, LDL-C was significantly lower in the UC group than in the telemedicine group. Plasma renin activity was not significantly different between the 2 groups at baseline, but significantly increased at the endpoint only in the UC group. The endpoint plasma renin activity was not significantly different between the 2 groups. Other laboratory data were not significantly different between the 2 groups.

### Prescription Data for Antihypertensive Medications

Percentages of antihypertensive treatment are shown in Table 5.

**Table 5:** Prescription data.

	Usual care group	Telemedicine group	
	Preintervention (n=48), n (%)	Preintervention (n=49), n (%)	Postintervention (n=48), n (%)
No medication	29 (60.0)	26 (54.2)	7 (14.6)
Calcium channel blocker only	11 (23.0)	13 (27.1)	8 (16.7)
Angiotensin II receptor blocker only	0 (0)	2 (4.2)	3 (6.3)
Mineralocorticoid receptor blocker only	2 (4.0)	0 (0)	3 (6.3)
Angiotensin II receptor blocker/angiotensin converting enzyme inhibitor + calcium channel blocker	5 (10.0)	7 (14.6)	22 (45.8)
Mineralocorticoid receptor blocker + calcium channel blocker	1 (2.0)	1 (2.1)	3 (6.3)
Angiotensin II receptor blocker + diuretic	0 (0)	0 (0)	0 (0)
Angiotensin II receptor blocker/angiotensin converting enzyme inhibitor + calcium channel blocker + diuretic	0 (0)	0 (0)	2 (4.2)

### Dropout Rates and Adverse Events

Of the 50 and 49 participants allocated to the UC and telemedicine groups, respectively, we assessed 46 from the UC group and 48 from the telemedicine group. In the UC group, a participant requested telemedicine, another did not meet the inclusion criteria, and another was lost to follow-up; hence, all 3 were excluded from the study. One participant from the UC group experienced a mild subarachnoid hemorrhage with no neurological deficits or hospitalization and dropped out of the study. In the telemedicine group, 1 participant experienced angina pectoris and discontinued the intervention. Medication-related complaints upon initiation or change of antihypertensive drugs included urticaria (n=1) and concerns of having considerably low BP (n=3). No discontinuation owing to drug side effects or difficulty using the telemedicine interface was recorded.

## Discussion

### Principal Findings

This study, for the first time in Japan, conducted hypertension treatment using telemonitoring and telemedicine without face-to-face visits for 1 year and revealed 2 major findings. First, telemedicine without actual office visits was determined to be relatively safe in managing hypertension for 1 year. Second, the telemedicine group achieved a lower BP than the UC group. Although the BP difference from baseline was not significantly different between the groups, the telemedicine group demonstrated a reduction in SBP of 9.2 mmHg, whereas the reduction in the UC group was 5.4 mmHg.

In a previous study that investigated the safety of telemedicine without office visits, the number of adverse events was not significantly different from that of UC. In the Telemonitoring and Self-management of Hypertension (TASMINH2) Trial, the frequency of side effects, such as swelling of legs, stiff joints, fatigue, and cough, was similar between the telemedicine and UC groups [5]. In our study, 2 patients dropped out owing to cardiovascular events during the study: 1 from the telemedicine group for new-onset angina pectoris and 1 from the UC group for subarachnoid hemorrhage. No significant difference in the laboratory findings was observed between the 2 groups at the end of the study, except for lower LDL-C in the UC group. Medication-related complaints were successfully managed through web-based consultations. The results of this and previous studies suggest that telemedicine is reasonably safe for use in controlling BP in uncomplicated hypertension.

Superior BP control in the telemedicine group than that in the UC group could be attributed to several factors, one being the intensity of the intervention. Sheppard et al [8] demonstrated that intense interventions, such as pharmacotherapeutic intervention managed by a pharmacist, with frequent telemonitoring, were more effective than low-intensity interventions, such as telemonitoring only, in patients with obesity. In this study, the telemedicine group received team-based care from physicians specializing in hypertension, and the participants were able to ask questions using the app. Therefore, the effects of antihypertensive intervention could have been enhanced by the more intensive interventions and greater expertise that the telemedicine group received. Several studies found that telemonitoring of BP improves BP control [3,4]. On the other hand, other studies showed that telemonitoring by itself does not significantly improve it [9]. Pellegrini et al [10] recently reported that self-monitoring of BP at home with co-interventions, such as telecounseling, favorably controlled BP more than self-monitoring of BP alone did. Nevertheless, regardless of their effects on BP control, advances in internet technology and data technology should accelerate the dissemination of BP telemonitoring and telemedicine, which would make retrospective analysis of BP management interventions in the future much easier than those in the past, when such analysis depended on a paper-based approach. In our previous study, an electronic sphygmomanometer with an automated 3G data transfer and room temperature monitor was used for daily SMBP [11]. BP variability based on SMBP can predict cardiovascular outcome in patients with hypertension [12,13]. Room temperature at the time of SMBP significantly correlates with variability of SBP [14,15]. Therefore, an HBP monitor equipped with various sensors, serving as the “Internet of Things,” may be used to monitor environmental conditions to maintain the optimal BP in future studies.

Regarding adherence, the frequency of HBP measurements was much higher in the telemedicine group than in the UC group. We do not have data on medication adherence, but Ogedegbe and Schoenthaler [16] demonstrated that HBP self-monitoring significantly improved medication adherence and reported that the telemedicine group self-monitored their BP more frequently.

### Limitations

This study has several limitations. First, this study was limited by its design in that the physicians’ level of expertise differed for the telemedicine and UC groups. Second, although the rate of treated hypertension at baseline was not different between the groups, changes in prescription were precisely tracked only in the telemedicine group. Third, the number of visits for the UC group was not determined, and the average number of visits could not be compared. In Japan, regular face-to-face visits for hypertension management in clinics generally occur at 2- to 8-week intervals. Fourth, because the study was terminated prematurely, it did not reach its intended sample size, making the power of the study insufficient for some analyses. Nevertheless, our finding of a significantly lower HBP at the end of the study period in the telemedicine group despite a smaller-than-intended sample size is promising for the improvement of BP control through telemedicine. Fifth (related to the first limitation), although the participants in the UC group were referred to primary care physicians with a letter asking them to target an HBP of less than 135/85 mmHg, it is not clear whether the physicians actually adhered to this request [17]. Although this study cannot delineate the effects of telemonitoring, telemedicine, and specialist intervention compared with UC, ours is a real-world study providing pilot data on hypertension telemedicine in Japan, which can be used to design future studies.

### Conclusions

Our results suggest that antihypertensive telemedicine using HBP telemonitoring and web-based video visits is safe. The telemedicine group of patients with uncomplicated hypertension achieved better BP control than the group assigned to conventional care. Further investigations are required to elucidate the benefits of telemedicine in treating hypertension on a larger scale.

## Appendix

Multimedia Appendix 1 CONSORT-eHEALTH checklist (V 1.6.1).

## Abbreviations

BP: blood pressure
DBP: diastolic blood pressure
HBP: home blood pressure
LDL-C: low-density-lipoprotein cholesterol
POATRAND: Paradigm of Antihypertensive Therapy Along With Telemedicine Randomized Trial
SBP: systolic blood pressure
SMBP: self-measurement of home blood pressure
UC: usual care
